# Autoimmune Hearing Loss: A Diagnostic Challenge

**DOI:** 10.3390/jcm11154601

**Published:** 2022-08-07

**Authors:** George Psillas, Grigorios George Dimas, Christos Savopoulos, Jiannis Constantinidis

**Affiliations:** 1 1st Otolaryngology Department, School of Medicine, Faculty of Health Sciences, Aristotle University of Thessaloniki, AHEPA Hospital, 54636 Thessaloniki, Greece; 2 1st Propedeutic Department of Internal Medicine, Faculty of Health Sciences, Aristotle University of Thessaloniki, AHEPA Hospital, 54636 Thessaloniki, Greece

Autoimmune hearing loss (AIHL) is a clinical disease and may involve the deposition of immune complexes in the labyrinth vessels, the activation of the complement system, the functional alteration in T-cell subpopulations, or an inflammation process in the inner ear [1]. The diagnosis AIHL is still unclear and there are no serologic or radiologic criteria for its identification. When initially described, AIHL was recognized as always being both bilateral and progressive [2]; however, AIHL may involve only one ear [3] and a significant proportion of patients with AIHL may experience a sudden onset of symptoms [4,5]. Moreover, positive response to steroid administration was considered as a clinical prerequisite for AIHL [2]; nevertheless, less than half of patients respond favorably to steroid therapy [5,6] and, over time, a progression of hearing loss has been observed.

Different pure-tone audiogram shapes in patients with AIHL have been recorded, including down-sloping (which is the most frequent), up-sloping, flat, cookie-bite, and inverse cookie-bite shapes; almost half of cases have moderate hearing loss [1,5]. AIHL is a long-standing disease with relapses and is more commonly observed after unilateral or bilateral sudden hearing loss [7].

Immunologic laboratory tests should be performed in every patient with suspected AIHL, such as complete blood count, erythrocyte sedimentation rate, antinuclear antibodies (ANA), antineutrophil cytoplasmic antibodies (ANCA), anticardiolipine/antiphospholipid antibodies, rheumatoid factor, immunoglobulines IgG, IgM, IgA, complement factors C3, C4, and anti-cochlin/anti-β-tubulin antibodies [8].

Genetic factors such as the human leukocyte antigen (HLA) system should also be investigated for AIHL. The haplotypes HLA B27, B35, B51, C4, C7, and A1-B8-DR3 have been reported as prognostic markers for the development of AIHL [9,10]. In a recent study [11], HLA DRB1*04 alleles were recorded at higher proportions compared with the healthy control group; HLA–DRB1*04 alleles were found to be strongly associated with Vogt–Koyanagi–Harada disease, which is an autoimmune disease that also affects the inner ear [12]. Moreover, HLA–DRB1*04 alleles, such as DRB1*0401, DRB1*0404, DRB1*0405, and DRB1*0408, have been reported to be highly expressed in patients suffering from rheumatoid arthritis [13]; finally, HLA DRB1*04, as a poor prognostic marker, was found at high frequency in patients with sudden sensorineural hearing loss and no satisfactory response to steroid treatment [14].

Presence of other autoimmune diseases should be investigated as potential clinical clues for AIHL in patients who have complained of progressive hearing loss or recurrent sudden hearing loss. Hashimoto’s thyroiditis is an autoimmune disorder that can cause hypothyroidism and in which significant increases in thyroid peroxidase antibody (anti-TPO) and thyroglobuline antibody (anti-Tg) have been found in a patient with AIHL [15]. In our study [5], we noted that Hashimoto’s thyroiditis was very commonly associated with patients suffering from AIHL; 13 (24.5%) out of 53 patients with AIHL had concomitant Hashimoto’s thyroiditis. It has been shown that, in Hashimoto’s thyroiditis, the number of T-regulatory (Treg) cells are reduced, and their function is defective, resulting in their limited downregulating effect against the ongoing autoimmune process and leading to extensive defects in the inner ear [16]. Close monitoring of hearing is recommended in the treatment of these patients with Hashimoto’s thyroiditis using a thorough audiometric test battery at least once a year.

In conclusion, this Special Issue gives new insight into the immune-mediated pathophysiologic mechanisms involving the inner ear. Specialized laboratory tests and an extended audiological test battery to identify underlying autoimmune process will be reported. Management of AIHL associated with other systemic autoimmune disorders will be discussed and new therapeutic protocols will be evaluated. Multicentric studies and collaboration are required to establish diagnostic criteria and therapeutic guidelines. Assessing the current knowledge and elucidating the effect of autoimmunity on the cochlea will help us to promote our understanding of this complex clinical disorder.

## Data Availability

The data presented in this study is available upon request from the corresponding author.
